# Supplementary material for: Pyruvate Dehydrogenase Kinase 1 inhibition mediated oxidative phosphorylation enhancement in cartilage promotes osteoarthritis progression
Source: BMC Musculoskelet Disord. 2023 Jul 20;24:597. doi: 10.1186/s12891-023-06585-6 (PMC10357736; doi:10.1186/s12891-023-06585-6)
Supplement: Supplementary file 1 — Additional file 1. [file 12891_2023_6585_MOESM1_ESM.zip › KEGG.Gsea.1653622432464/KEGG_ACUTE_MYELOID_LEUKEMIA.html]

Details for gene set KEGG\_ACUTE\_MYELOID\_LEUKEMIA[GSEA]

|  || Dataset | 55457\_collapsed\_to\_symbols.cls.cls #OA\_versus\_normal.cls.cls #OA\_versus\_normal\_repos |
| Phenotype | cls.cls#OA\_versus\_normal\_repos |
| Upregulated in class | normal |
| GeneSet | KEGG\_ACUTE\_MYELOID\_LEUKEMIA |
| Enrichment Score (ES) | -0.3834415 |
| Normalized Enrichment Score (NES) | -1.5038005 |
| Nominal p-value | 0.017331023 |
| FDR q-value | 0.20286956 |
| FWER p-Value | 0.901 |
Table: GSEA Results Summary

  

Fig 1: Enrichment plot: KEGG\_ACUTE\_MYELOID\_LEUKEMIA      
 Profile of the Running ES Score & Positions of GeneSet Members on the Rank Ordered List

  

| SYMBOL | TITLE | RANK IN GENE LIST | RANK METRIC SCORE | RUNNING ES | CORE ENRICHMENT || 1 | MAPK3 | mitogen-activated protein kinase 3 [Source:HGNC Symbol;Acc:HGNC:6877] | 757 | 0.579 | -0.0228 | No |
| 2 | AKT1 | AKT serine/threonine kinase 1 [Source:HGNC Symbol;Acc:HGNC:391] | 1192 | 0.478 | -0.0267 | No |
| 3 | CEBPA | CCAAT enhancer binding protein alpha [Source:HGNC Symbol;Acc:HGNC:1833] | 1750 | 0.392 | -0.0456 | No |
| 4 | KIT | "KIT proto-oncogene, receptor tyrosine kinase [Source:HGNC Symbol;Acc:HGNC:6342]" | 2117 | 0.348 | -0.0524 | No |
| 5 | PIK3CB | "phosphatidylinositol-4,5-bisphosphate 3-kinase catalytic subunit beta [Source:HGNC Symbol;Acc:HGNC:8976]" | 2186 | 0.340 | -0.0365 | No |
| 6 | PIM2 | "Pim-2 proto-oncogene, serine/threonine kinase [Source:HGNC Symbol;Acc:HGNC:8987]" | 3623 | 0.192 | -0.1363 | No |
| 7 | BAD | BCL2 associated agonist of cell death [Source:HGNC Symbol;Acc:HGNC:936] | 3788 | 0.178 | -0.1380 | No |
| 8 | RELA | "RELA proto-oncogene, NF-kB subunit [Source:HGNC Symbol;Acc:HGNC:9955]" | 3942 | 0.161 | -0.1398 | No |
| 9 | NFKB1 | nuclear factor kappa B subunit 1 [Source:HGNC Symbol;Acc:HGNC:7794] | 4626 | 0.102 | -0.1866 | No |
| 10 | FLT3 | fms related receptor tyrosine kinase 3 [Source:HGNC Symbol;Acc:HGNC:3765] | 4657 | 0.099 | -0.1828 | No |
| 11 | TCF7L2 | transcription factor 7 like 2 [Source:HGNC Symbol;Acc:HGNC:11641] | 5034 | 0.070 | -0.2077 | No |
| 12 | RPS6KB2 | ribosomal protein S6 kinase B2 [Source:HGNC Symbol;Acc:HGNC:10437] | 5496 | 0.033 | -0.2415 | No |
| 13 | RUNX1T1 | RUNX1 partner transcriptional co-repressor 1 [Source:HGNC Symbol;Acc:HGNC:1535] | 5503 | 0.032 | -0.2400 | No |
| 14 | STAT5B | signal transducer and activator of transcription 5B [Source:HGNC Symbol;Acc:HGNC:11367] | 5554 | 0.028 | -0.2421 | No |
| 15 | GRB2 | growth factor receptor bound protein 2 [Source:HGNC Symbol;Acc:HGNC:4566] | 5697 | 0.016 | -0.2521 | No |
| 16 | EIF4EBP1 | eukaryotic translation initiation factor 4E binding protein 1 [Source:HGNC Symbol;Acc:HGNC:3288] | 5871 | 0.004 | -0.2654 | No |
| 17 | IKBKB | inhibitor of nuclear factor kappa B kinase subunit beta [Source:HGNC Symbol;Acc:HGNC:5960] | 5914 | 0.001 | -0.2686 | No |
| 18 | RAF1 | "Raf-1 proto-oncogene, serine/threonine kinase [Source:HGNC Symbol;Acc:HGNC:9829]" | 6187 | -0.019 | -0.2885 | No |
| 19 | PML | PML nuclear body scaffold [Source:HGNC Symbol;Acc:HGNC:9113] | 6421 | -0.037 | -0.3044 | No |
| 20 | MAPK1 | mitogen-activated protein kinase 1 [Source:HGNC Symbol;Acc:HGNC:6871] | 6853 | -0.068 | -0.3337 | No |
| 21 | STAT5A | signal transducer and activator of transcription 5A [Source:HGNC Symbol;Acc:HGNC:11366] | 6988 | -0.079 | -0.3392 | No |
| 22 | NRAS | "NRAS proto-oncogene, GTPase [Source:HGNC Symbol;Acc:HGNC:7989]" | 7300 | -0.102 | -0.3570 | No |
| 23 | TCF7L1 | transcription factor 7 like 1 [Source:HGNC Symbol;Acc:HGNC:11640] | 7383 | -0.109 | -0.3566 | No |
| 24 | PIK3CD | "phosphatidylinositol-4,5-bisphosphate 3-kinase catalytic subunit delta [Source:HGNC Symbol;Acc:HGNC:8977]" | 7405 | -0.111 | -0.3513 | No |
| 25 | LEF1 | lymphoid enhancer binding factor 1 [Source:HGNC Symbol;Acc:HGNC:6551] | 7467 | -0.116 | -0.3488 | No |
| 26 | CCND1 | cyclin D1 [Source:HGNC Symbol;Acc:HGNC:1582] | 7512 | -0.119 | -0.3448 | No |
| 27 | PIK3CA | "phosphatidylinositol-4,5-bisphosphate 3-kinase catalytic subunit alpha [Source:HGNC Symbol;Acc:HGNC:8975]" | 7733 | -0.136 | -0.3534 | No |
| 28 | PIK3CG | "phosphatidylinositol-4,5-bisphosphate 3-kinase catalytic subunit gamma [Source:HGNC Symbol;Acc:HGNC:8978]" | 7953 | -0.152 | -0.3610 | No |
| 29 | HRAS | "HRas proto-oncogene, GTPase [Source:HGNC Symbol;Acc:HGNC:5173]" | 7977 | -0.153 | -0.3532 | No |
| 30 | AKT3 | AKT serine/threonine kinase 3 [Source:HGNC Symbol;Acc:HGNC:393] | 8008 | -0.156 | -0.3458 | No |
| 31 | SOS2 | SOS Ras/Rho guanine nucleotide exchange factor 2 [Source:HGNC Symbol;Acc:HGNC:11188] | 8058 | -0.160 | -0.3397 | No |
| 32 | ARAF | "A-Raf proto-oncogene, serine/threonine kinase [Source:HGNC Symbol;Acc:HGNC:646]" | 8092 | -0.163 | -0.3321 | No |
| 33 | BRAF | "B-Raf proto-oncogene, serine/threonine kinase [Source:HGNC Symbol;Acc:HGNC:1097]" | 8171 | -0.169 | -0.3276 | No |
| 34 | STAT3 | signal transducer and activator of transcription 3 [Source:HGNC Symbol;Acc:HGNC:11364] | 8453 | -0.190 | -0.3376 | No |
| 35 | CCNA1 | cyclin A1 [Source:HGNC Symbol;Acc:HGNC:1577] | 8584 | -0.200 | -0.3353 | No |
| 36 | PIK3R3 | phosphoinositide-3-kinase regulatory subunit 3 [Source:HGNC Symbol;Acc:HGNC:8981] | 8801 | -0.217 | -0.3386 | No |
| 37 | PIK3R5 | phosphoinositide-3-kinase regulatory subunit 5 [Source:HGNC Symbol;Acc:HGNC:30035] | 8804 | -0.217 | -0.3252 | No |
| 38 | SPI1 | Spi-1 proto-oncogene [Source:HGNC Symbol;Acc:HGNC:11241] | 9012 | -0.232 | -0.3268 | No |
| 39 | RUNX1 | RUNX family transcription factor 1 [Source:HGNC Symbol;Acc:HGNC:10471] | 9063 | -0.237 | -0.3159 | No |
| 40 | MAP2K2 | mitogen-activated protein kinase kinase 2 [Source:HGNC Symbol;Acc:HGNC:6842] | 9932 | -0.315 | -0.3638 | Yes |
| 41 | JUP | junction plakoglobin [Source:HGNC Symbol;Acc:HGNC:6207] | 10101 | -0.331 | -0.3562 | Yes |
| 42 | RARA | retinoic acid receptor alpha [Source:HGNC Symbol;Acc:HGNC:9864] | 10141 | -0.335 | -0.3383 | Yes |
| 43 | MAP2K1 | mitogen-activated protein kinase kinase 1 [Source:HGNC Symbol;Acc:HGNC:6840] | 10197 | -0.340 | -0.3214 | Yes |
| 44 | RPS6KB1 | ribosomal protein S6 kinase B1 [Source:HGNC Symbol;Acc:HGNC:10436] | 10224 | -0.343 | -0.3019 | Yes |
| 45 | ZBTB16 | zinc finger and BTB domain containing 16 [Source:HGNC Symbol;Acc:HGNC:12930] | 10246 | -0.346 | -0.2820 | Yes |
| 46 | KRAS | "KRAS proto-oncogene, GTPase [Source:HGNC Symbol;Acc:HGNC:6407]" | 10544 | -0.377 | -0.2815 | Yes |
| 47 | PIK3R2 | phosphoinositide-3-kinase regulatory subunit 2 [Source:HGNC Symbol;Acc:HGNC:8980] | 10956 | -0.425 | -0.2870 | Yes |
| 48 | IKBKG | inhibitor of nuclear factor kappa B kinase regulatory subunit gamma [Source:HGNC Symbol;Acc:HGNC:5961] | 10986 | -0.428 | -0.2625 | Yes |
| 49 | PIK3R1 | phosphoinositide-3-kinase regulatory subunit 1 [Source:HGNC Symbol;Acc:HGNC:8979] | 11099 | -0.443 | -0.2435 | Yes |
| 50 | PPARD | peroxisome proliferator activated receptor delta [Source:HGNC Symbol;Acc:HGNC:9235] | 11301 | -0.471 | -0.2298 | Yes |
| 51 | CHUK | component of inhibitor of nuclear factor kappa B kinase complex [Source:HGNC Symbol;Acc:HGNC:1974] | 11624 | -0.521 | -0.2223 | Yes |
| 52 | SOS1 | SOS Ras/Rac guanine nucleotide exchange factor 1 [Source:HGNC Symbol;Acc:HGNC:11187] | 12041 | -0.603 | -0.2170 | Yes |
| 53 | MTOR | mechanistic target of rapamycin kinase [Source:HGNC Symbol;Acc:HGNC:3942] | 12047 | -0.605 | -0.1796 | Yes |
| 54 | PIM1 | "Pim-1 proto-oncogene, serine/threonine kinase [Source:HGNC Symbol;Acc:HGNC:8986]" | 12500 | -0.748 | -0.1681 | Yes |
| 55 | TCF7 | transcription factor 7 [Source:HGNC Symbol;Acc:HGNC:11639] | 12628 | -0.813 | -0.1272 | Yes |
| 56 | MYC | "MYC proto-oncogene, bHLH transcription factor [Source:HGNC Symbol;Acc:HGNC:7553]" | 12801 | -0.970 | -0.0800 | Yes |
| 57 | AKT2 | AKT serine/threonine kinase 2 [Source:HGNC Symbol;Acc:HGNC:392] | 12896 | -1.404 | 0.0003 | Yes |
Table: GSEA details [plain text format]

  

Fig 2: KEGG\_ACUTE\_MYELOID\_LEUKEMIA      
 Blue-Pink O' Gram in the Space of the Analyzed GeneSet

  

Fig 3: KEGG\_ACUTE\_MYELOID\_LEUKEMIA: Random ES distribution      
 Gene set null distribution of ES for **KEGG\_ACUTE\_MYELOID\_LEUKEMIA**
